# Supplementary material for: Contrasting gene flow at different spatial scales revealed by genotyping-by-sequencing in Isocladus armatus, a massively colour polymorphic New Zealand marine isopod
Source: PeerJ. 2018 Aug 22;6:e5462. doi: 10.7717/peerj.5462 (PMC6109376; doi:10.7717/peerj.5462)
Supplement: Table S1 [file peerj-06-5462-s001.docx]

| Filter applied | Number of putative SNPs |
| --- | --- |
|  | 26,215 |
| SNPs with a replication average < 0.99 | 24,550 |
| SNPs with frequency of heterozygotes > 0.5 | 24,491 |
| SNPs with average read depth < 5X for both alleles | 20,189 |
| SNPs with a Call Rate < 0.8 | 17,898 |
| SNPs with MAF < 0.05 | 6,567 |
| 1 SNP per read with greatest H | 5,236 |
| SNPs out of HWE in at least 1 population | 4,002 |
| SNPs deemed under selection | 3,892 |
