# Supplementary material for: Contrasting gene flow at different spatial scales revealed by genotyping-by-sequencing in Isocladus armatus, a massively colour polymorphic New Zealand marine isopod
Source: PeerJ. 2018 Aug 22;6:e5462. doi: 10.7717/peerj.5462 (PMC6109376; doi:10.7717/peerj.5462)
Supplement: Table S2 — Analyses were run with all three populations, and with only the two North Island (Hatfield’s Beach and Stanmore Bay) populations. Loci are listed in order of decreasing significance. [file peerj-06-5462-s002.docx]

| All populations | | North Island only | |
| --- | --- | --- | --- |
| Bayescan | Arlequin | Bayescan | Arlequin |
| 594 | 4901 | 4901 | 4901 |
| 4901 | 594 | 594 | 594 |
| 514 | 4676 | 2075 | 3414 |
| 4676 | 104 | 3414 | 2075 |
|  |  | 4161 | 4161 |
|  |  | 2746 | 2746 |
