# Supplementary figures and images for: Contrasting gene flow at different spatial scales revealed by genotyping-by-sequencing in Isocladus armatus, a massively colour polymorphic New Zealand marine isopod
Source: PeerJ. 2018 Aug 22;6:e5462. doi: 10.7717/peerj.5462 (PMC6109376; doi:10.7717/peerj.5462)

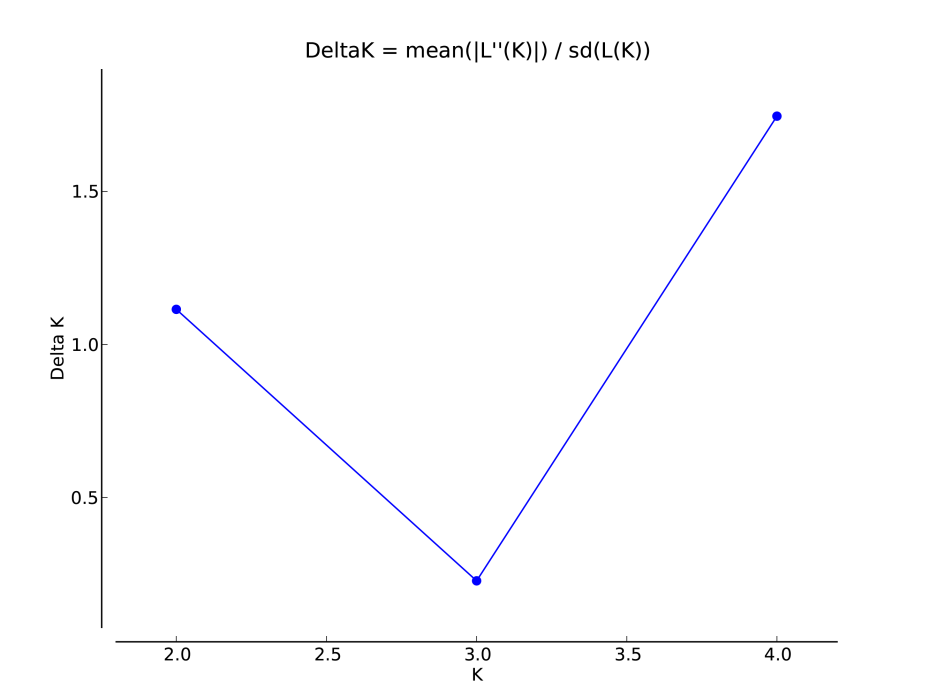

Supplement: Figure S1 [file peerj-06-5462-s003.png]

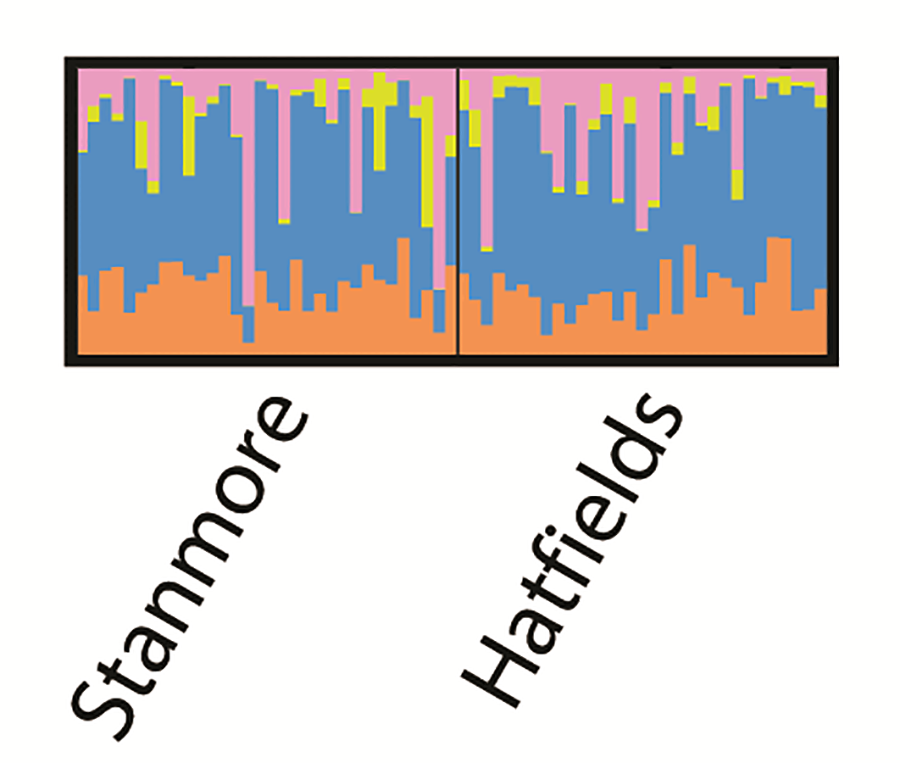

Supplement: Figure S2 — Colours indicate proportional membership to a genetic cluster summarised over 10 replicates in the program CLUMPP. Individuals are ordered by population of origin with vertical bars representing a single individual. [file peerj-06-5462-s004.png]

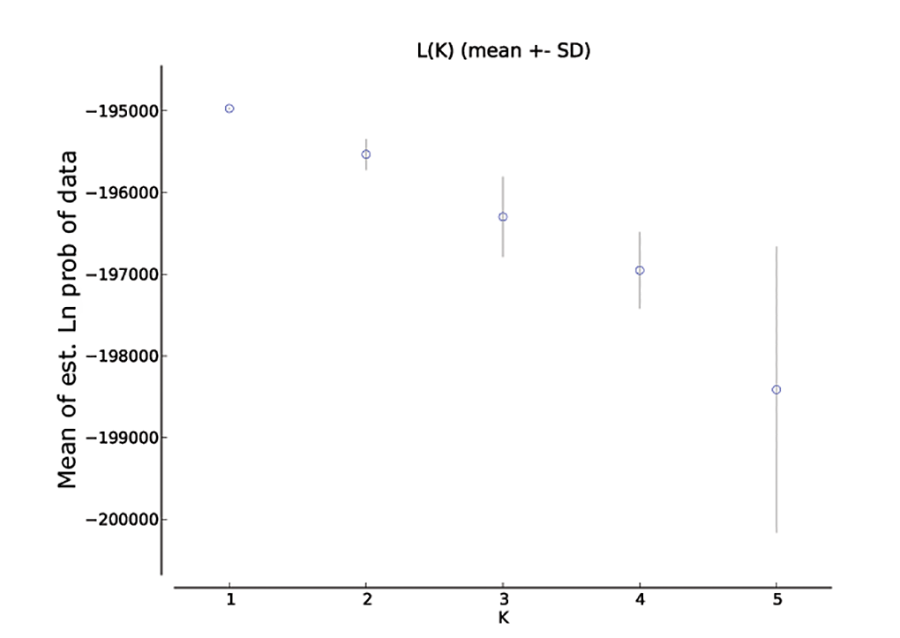

Supplement: Figure S3 [file peerj-06-5462-s005.png]

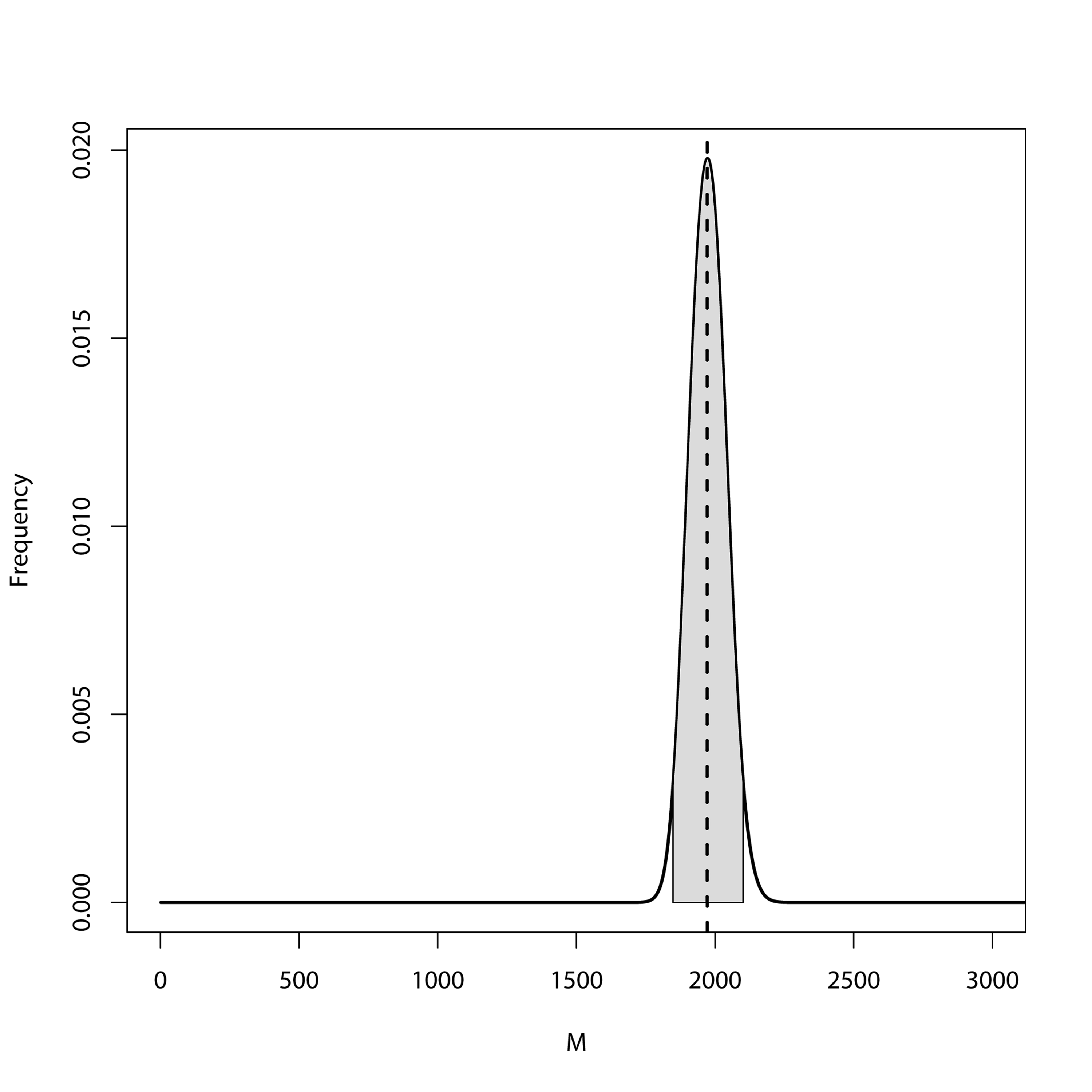

Supplement: Figure S4 — Grey shaded area shows the 95% HPD intervals. Dashed line represents the mode of the posterior. [file peerj-06-5462-s006.png]

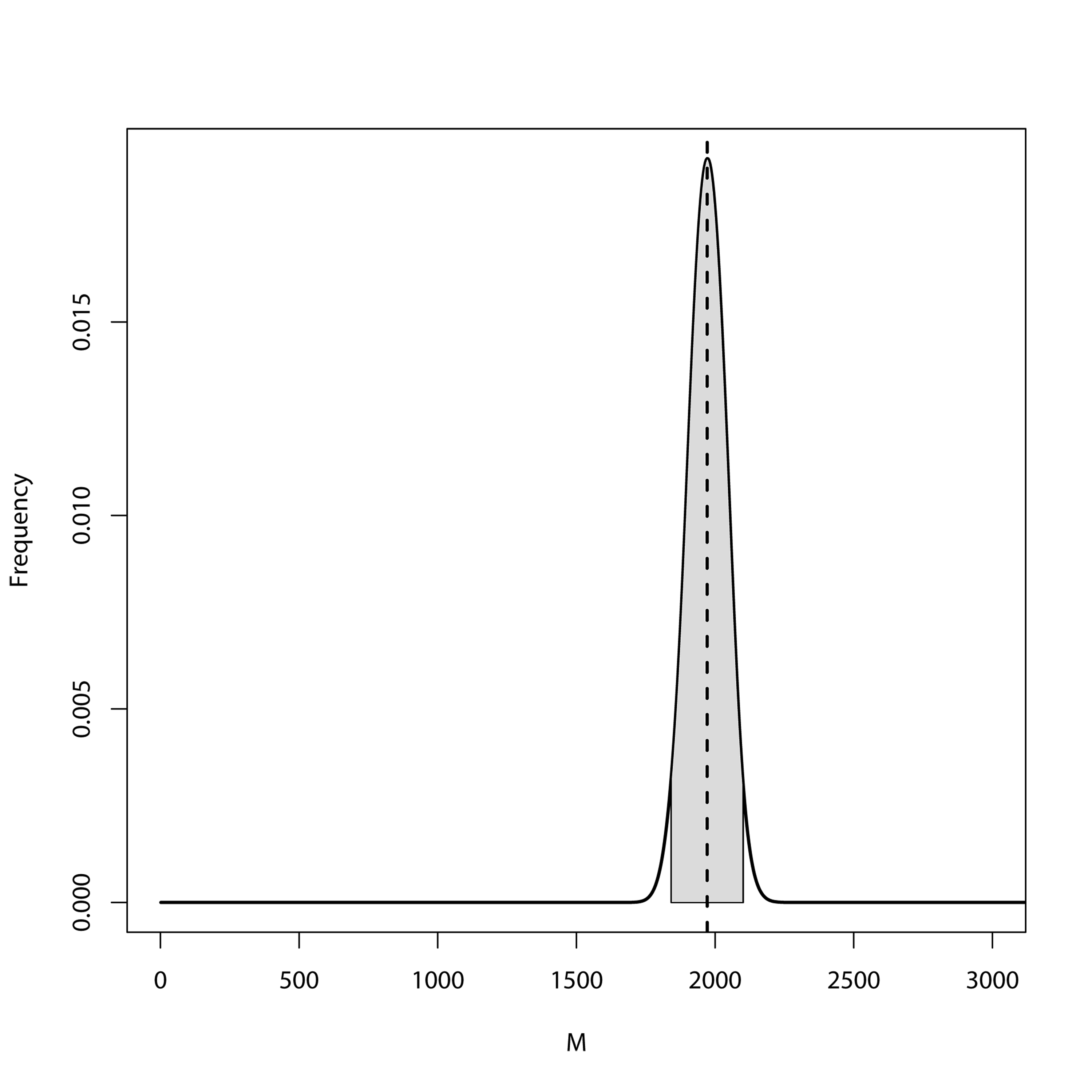

Supplement: Figure S5 — Grey shaded area shows the 95% HPD intervals. Dashed line represents the mode of the posterior. [file peerj-06-5462-s007.png]

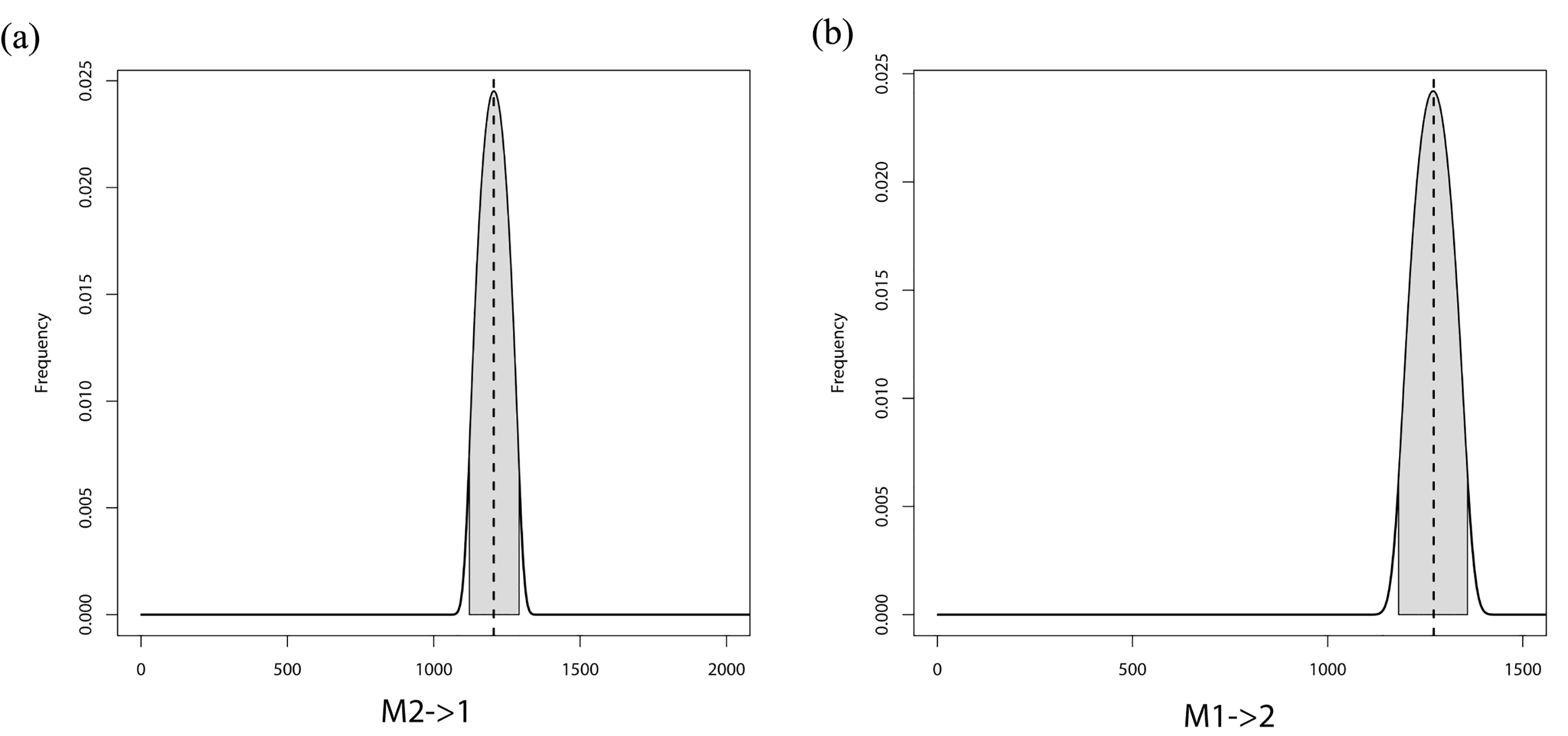

Supplement: Figure S6 — Grey shaded area shows the 95% HPD intervals. Dashed line represents the mode of the posterior. [file peerj-06-5462-s008.png]

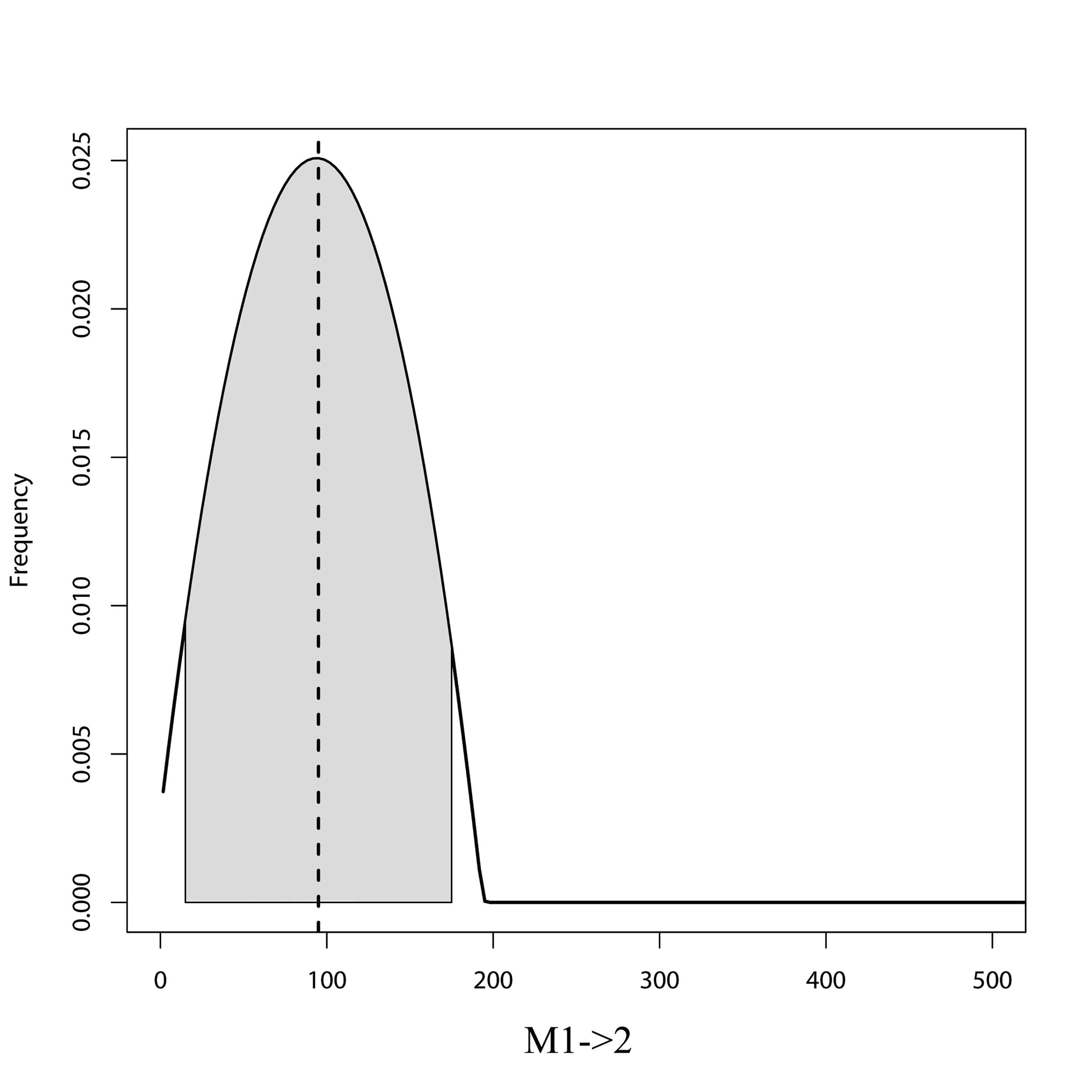

Supplement: Figure S7 — Grey shaded area shows the 95% HPD intervals. Dashed line represents the mode of the posterior. [file peerj-06-5462-s009.png]

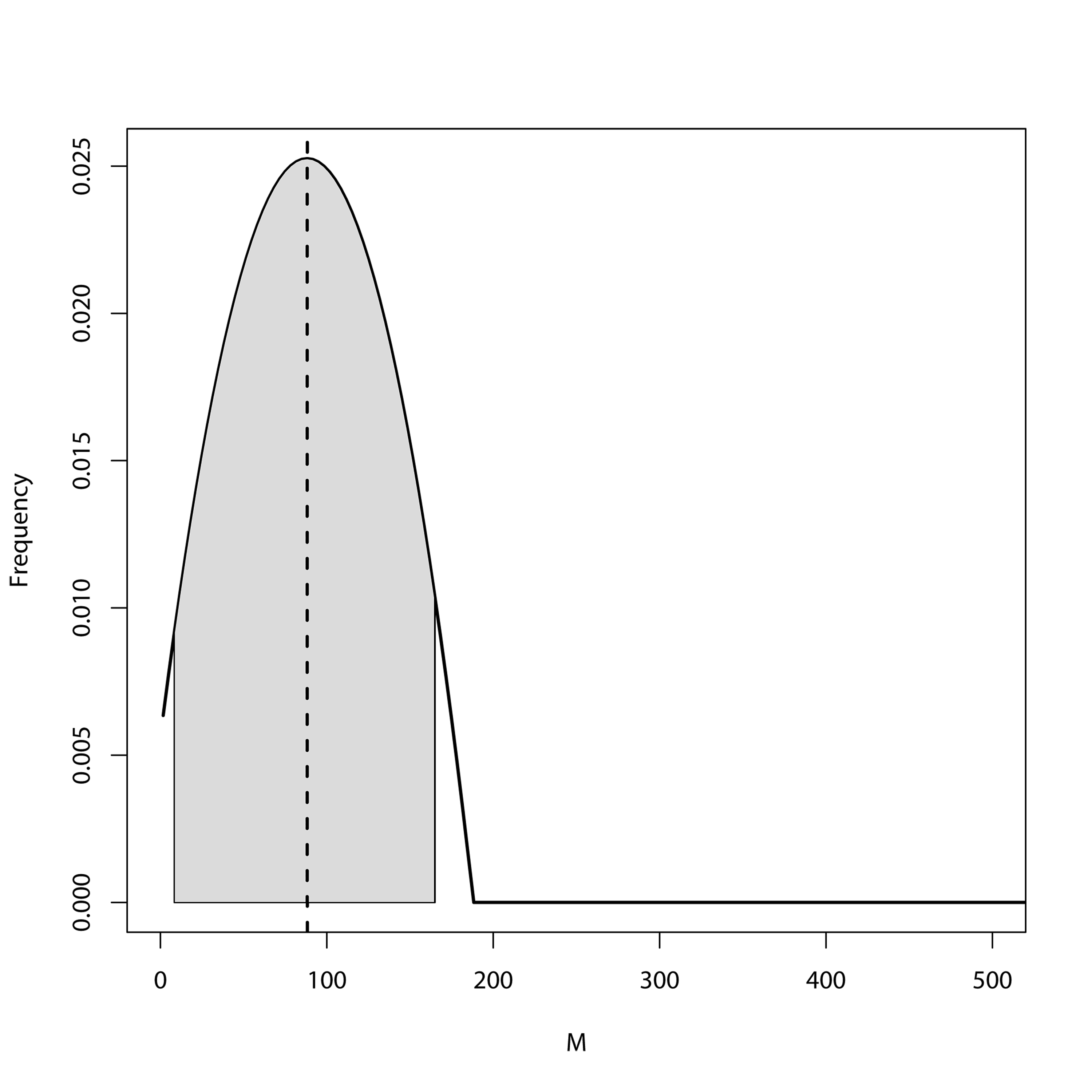

Supplement: Figure S8 — Grey shaded area shows the 95% HPD intervals. Dashed line represents the mode of the posterior. [file peerj-06-5462-s010.png]

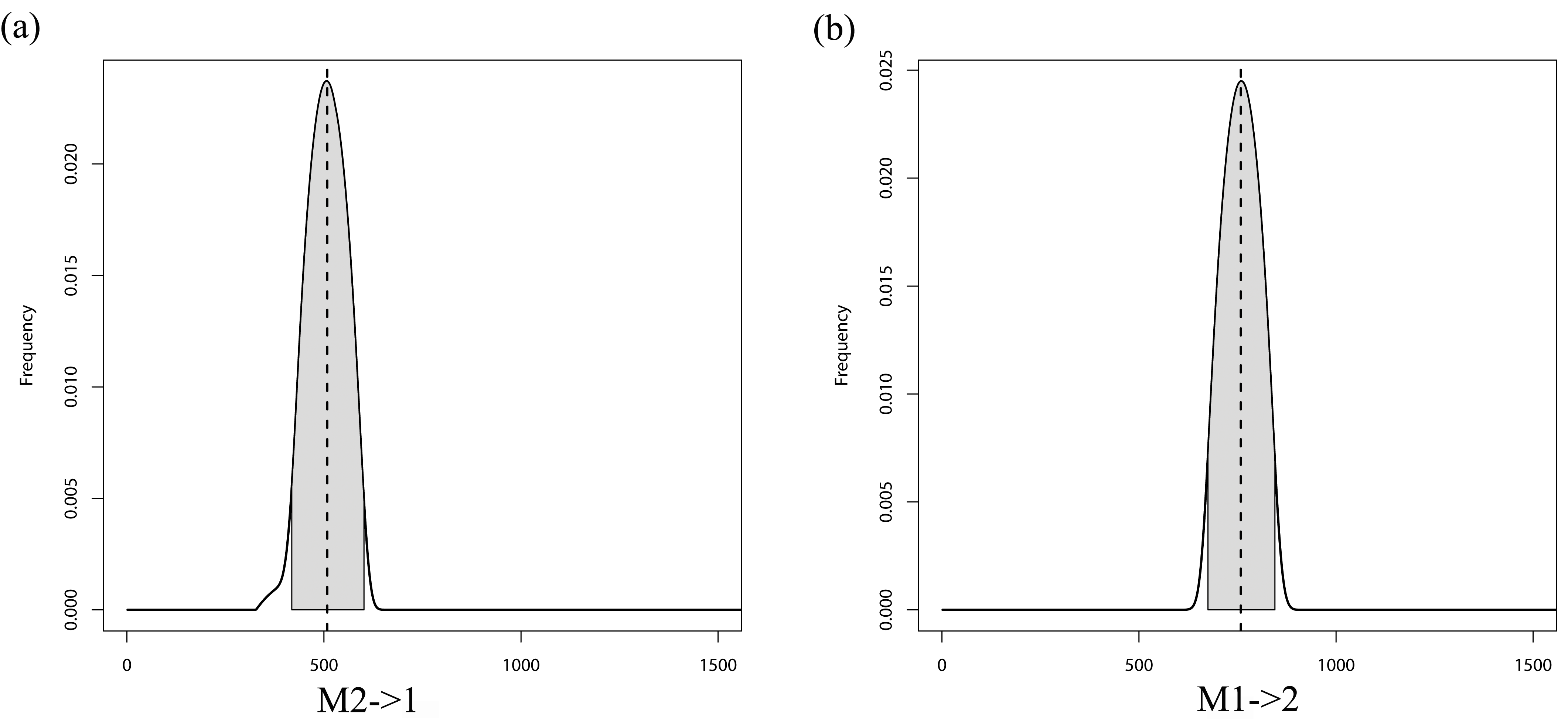

Supplement: Fugure S9 — Grey shaded area shows the 95% HPD intervals. Dashed line represents the mode of the posterior. [file peerj-06-5462-s011.png]

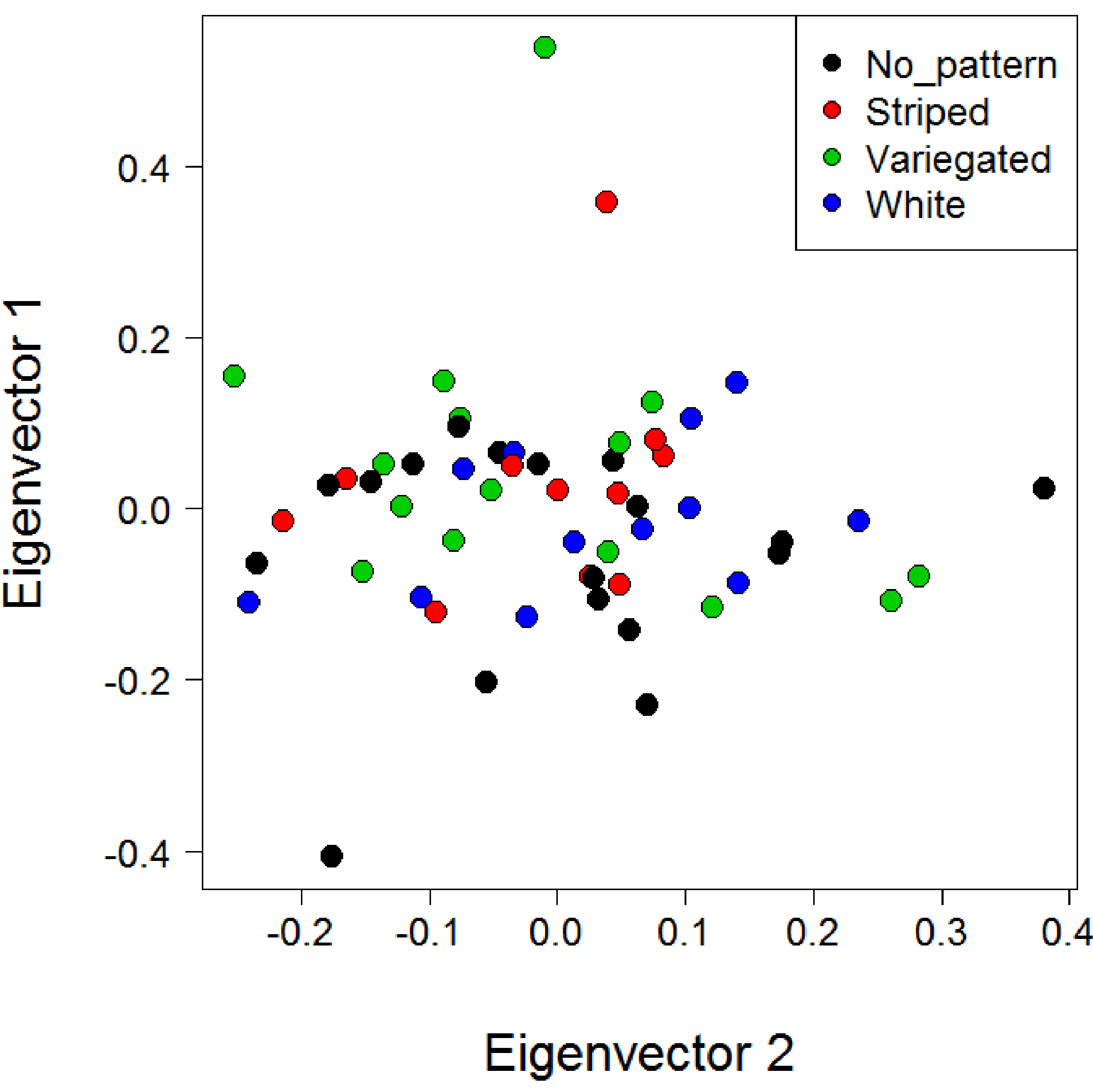

Supplement: Figure S10 — The first and second principal coordinates explain 2.1% and 2.1% of the variance respectively. [file peerj-06-5462-s012.png]
